# Identification of metabolites from the gut microbiota in hypertension via network pharmacology and molecular docking

**DOI:** 10.1186/s40643-024-00815-y

**Published:** 2024-10-21

**Authors:** Wenjie Zhang, Yinming Zhang, Jun Li, Jiawei Tang, Ji Wu, Zicong Xie, Xuanchun Huang, Shiyi Tao, Tiantian Xue

**Affiliations:** 1 Department of Cardiology, Guang’anmen Hospital, China Academy of Chinese Medical Sciences, No.5 Beixiange, Xicheng District, Beijing, 100053 China; 2 Graduate School, Beijing University of Chinese Medicine, Beijing, China; 3 Department of Emergency, Yankuang New Journey General Hospital, Zoucheng, Shandong Province China; 4 School of Computer Science, Beijing University of Posts and Telecommunications, Beijing, China

**Keywords:** Hypertension, Gut microbiota, Metabolite, Network pharmacology, Molecular docking, Equol

## Abstract

Hypertension is the most prevalent cardiovascular disease, affecting one-third of adults. All antihypertensive drugs have potential side effects. Gut metabolites influence hypertension. The objective of this study was to identify antihypertensive gut metabolites through network pharmacology and molecular docking techniques and to validate their antihypertensive mechanisms via in vitro experiments. A total of 10 core antihypertensive targets and 18 gut metabolites that act on hypertension were identified. Four groups of protein metabolites, namely, CXCL8-baicalein, CXCL8-baicalin, CYP1A1-urolithin A, and PTGS2-equol, which have binding energies of − 7.7, − 8.5, − 7.2, and − 8.8 kcal-mol^−1^, respectively, were found to have relatively high affinities. Based on its drug-likeness properties in silico and toxicological properties, equol was identified as a potential antihypertensive metabolite. On the basis of the results of network pharmacology and molecular docking, equol may exert antihypertensive effects by regulating the IL-17 signaling pathway and PTGS2. A phenylephrine-induced H9c2 cell model was subsequently utilized to verify that equol inhibits cell hypertrophy (P < 0.05) by inhibiting the IL-17 signaling pathway and PTGS2 (P < 0.05). This study demonstrated that equol has the potential to be developed as a novel therapeutic agent for the treatment of hypertension.

**Graphical Abstract:**

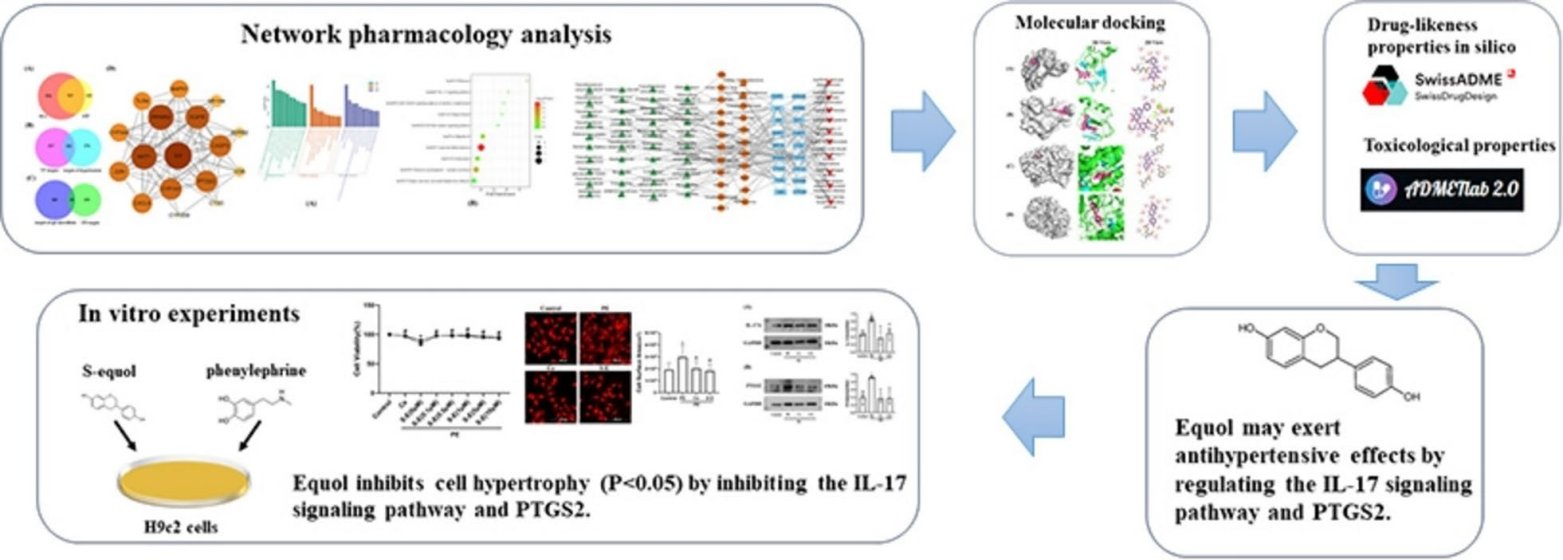

**Supplementary Information:**

The online version contains supplementary material available at 10.1186/s40643-024-00815-y.

## Introduction

Hypertension is the most prevalent cardiovascular disease (CVD) and increases the risk of stroke, coronary artery disease, heart failure, and chronic kidney disease (Zhou et al. 2021). The World Health Organization states that hypertension affects approximately one-third of adults globally (NCD Risk Factor Collaboration, 2021). Furthermore, in 2019, hypertension contributed to 10.8 million deaths (GBD 2019 Risk Factors Collaborators 2020), thus emerging as a primary risk factor for mortality worldwide. Modern medicine controls hypertension incidence through lifestyle interventions (Williams et al. 2018), such as weight loss (Fu et al. 2020), restricting sodium intake (Graudal et al. 2019; Huang et al. 2020), augmenting daily physical activities (Liu et al. 2017a, b; Rijal et al. 2023), maintaining moderate alcohol consumption (Biddinger et al. 2022; Roerecke et al. 2018), smoking cessation (Cheung et al. 2021; Hartmann-Boyce et al. 2021), increasing stress (Liu et al. 2017a, b; Albus et al. 2019), and improving the living environment (Wojciechowska et al. 2022). In cases where lifestyle interventions fail to control blood pressure, prompt initiation of antihypertensive medication is necessary (Mancia et al. 2023). However, all antihypertensive drugs have the potential for side effects (Laurent 2017), which probably contribute to non-adherence and discontinuation of treatment (Espeche et al. 2020; Rea et al. 2021). Therefore, safer and more effective hypertension therapies are urgently needed.

O'Donnell et al. reviewed the evidence that the gut microbiota influences hypertension (O'Donnell et al. 2023), with underlying mechanisms related to the generation of gut metabolites (Yang et al. 2023), specifically trimethylamine N-oxide (Jiang et al. 2021) and lipopolysaccharide (Sun et al. 2018), which affect hypertension. A metagenomic study revealed significantly reduced butyrate levels in the stools of patients with hypertension (Kim et al. 2018). Butyrate is a short-chain fatty acid (SCFA) that is known to lower blood pressure through various mechanisms, including vasodilation of peripheral blood vessels, protection of vascular endothelial function, attenuation of inflammatory responses, inhibition of the sympathetic nervous system, and renal protection (Hu et al. 2022).

Gut metabolites, as the body's own metabolites, have safety advantages over current antihypertensive drugs. Owing to the complex composition of gut metabolites, it is necessary to find suitable research methods to explore gut metabolites with antihypertensive effects. In addition, exploring hypertension from the perspective of gut metabolites may help elucidate the pathogenesis of hypertension and advance the prevention and treatment of hypertension. Network pharmacology and molecular docking techniques can be used to determine the potential mode of action of drugs and disease targets in silico (Kaur et al. 2019), providing ideas for the discovery of new antihypertensive drugs.

In this study, we first employed network pharmacology to identify the gut metabolites associated with hypertension. We then applied Gene Ontology (GO) (Gene Ontology Consortium 2019) and Kyoto Encyclopedia of Genes and Genomes (KEGG) (Kanehisa et al. 2017) enrichment analyses to identify the crucial signaling pathways and core targets of the gut metabolites affecting hypertension. Next, we used molecular docking technology to validate the interaction modes between potential metabolites and core targets. Finally, we verified the mechanism of action of potential metabolites and core targets in a cell model. This study aimed to identify gut metabolites with antihypertensive properties and provide new ideas for the treatment of hypertension.

## Methods and materials

### Selection of gut metabolites and targets

The gut metabolites and their targets were acquired from the gutMGene v1.0 database (http://bio-computing.hrbmu.edu.cn/gutmgene/accessed on April 14, 2024) (Cheng et al. 2022). The gut metabolites were retrieved via the simplified molecular-input line-entry system (SMILES) in PubChem (https://pubchem.ncbi.nlm.nih.gov/accessed on April 14, 2024), and the canonical SMILES of the metabolites were obtained. In the "*Homo sapiens*" setting, the targets of gut metabolites were predicted via the similarity ensemble approach (SEA) (https://sea.bkslab.org/accessed on April 16, 2024) (Keiser et al. 2007) and SwissTargetPrediction (STP) (http://swisstargetprediction.ch/accessed on April 18, 2024) (Gfeller et al. 2013). The DeepVenn platform (http://www.deepvenn.com/ accessed on April 20, 2024) (Hulsen 2022) was subsequently used to analyze the overlapping targets between the SEA and STP databases.

### Identification of the core targets of gut metabolites involved in hypertension

The hypertension targets were downloaded from GeneCards (https://www.genecards.org/accessed on April 18, 2024) (Stelzer et al. 2016), Online Mendelian Inheritance in Man (OMIM) (https://omim.org/accessed on April 18, 2024) (Hamosh et al. 2005), and the Therapeutic Target Database (TTD) (https://db.idrblab.net/ttd/accessed on April 18, 2024) (Zhou et al. 2024). Targets with scores greater than five were retained from the GeneCards database. The DeepVenn platform was used to intersect hypertension-related targets and gut metabolite targets to identify potential targets of gut metabolites that act on hypertension. Finally, the potential targets of the gut metabolites that act on hypertension and the targets of the gut metabolites were intersected to identify the core targets of the gut metabolites that act on hypertension.

### Construction of the protein‒protein interaction (PPI) network

Core targets were analyzed via STRING (https://string-db.org/accessed on April 22, 2024) (Szklarczyk et al. 2023), and subsequently, the "tsv" file was downloaded and imported into Cytoscape 3.10.1 software to construct the PPI network. The organism was designated "Homo sapiens", and the minimum required interaction score was established at "medium confidence (0.4)"; thereafter, the PPI network was constructed. The target exhibiting the highest value in the PPI network was deemed the central target for hypertension.

### Analysis of the GO and KEGG pathway enrichment of the gut metabolites

The core targets were imported into the DAVID platform (https://david.ncifcrf.gov/tools.jsp accessed on April 24, 2024) (Sherman et al. 2022) for GO and KEGG enrichment analysis, and the species was set to "*Homo sapiens*.” GO analysis included molecular function (MF), biological function (BF), and cellular component (CC) analyses. KEGG analysis was performed to identify the potential signaling pathways of gut metabolites that act on hypertension. Finally, visual analysis was performed via a bioinformatics platform (https://www.bioinformatics.com.cn/accessed on April 25, 2024) (Tang et al. 2023).

### Microbiota–metabolites–targets–signaling pathways (MMTS) network analysis

Cytoscape 3.10.1 software was used to construct MMTS network graphs and analyze the relationships among the gut microbiota, metabolites, targets, and signaling pathways. In the MMTS network diagram, each component or target is represented by a node, and the relationship between the component and target is represented by a connecting line.

### Docking testing of metabolites and targets for molecular

We downloaded the 3D structures of the metabolites from the PubChem database and saved them in "sdf" format. The name of the core target was determined via UniProt (https://www.uniprot.org/accessed on April 26, 2024), and the 3D structures of the core targets were downloaded from the RCSB Protein Data Bank (RCSB PDB) database (https://www.rcsb.org/accessed on April 26, 2024) and saved in "pdb" format. Open Babel 2.4.1 was applied to convert the "sdf" format of the metabolite to the "pdbqt" format. Ligands and receptors were prepared by dehydration and hydrogenation via AutoDockTools software. AutoDock Vina 1.1.2 (Trott and Olson 2010) was used to dock the metabolite target, and the results of the docking with lower binding energies were visualized via LigPlot (Laskowski and Swindells 2011) and PyMOL (Seeliger and de Groot 2010) software.

### Evaluation of drug-likeness properties

We used SwissAMDE (http://www.swissadme.ch/accessed on May 10, 2024) (Daina et al. 2017) and the literature to evaluate the drug-likeness properties of the metabolites. Metabolites are usually hydrophilic and have low bioavailability. Therefore, we determined their physical and chemical properties via silicon strategies.

### Toxicological evaluation by ADMETlab

Safety evaluation is essential for drug development. We identified eight toxicological parameters of metabolites via ADMETlab 2.0 (https://admetmesh.scbdd.com/accessed on May 10, 2024) (Xiong et al. 2021): hERG blockers (Wang et al. 2016), human hepatotoxicity (H-HT) (Mulliner et al. 2016), AMES toxicity (Xu et al. 2012), respiratory toxicity (Lei et al. 2017), rat oral acute toxicity (Roy et al. 2019), drug-induced liver injury (DILI) (Xu et al. 2015), skin sensitization (Alves et al. 2015), and lethal dose 50 (LD50) of acute toxicity (Lei et al. 2016).

### Cell culture and cell proliferation assay

H9c2 rat cardiomyoblast cells were purchased from Procell (CL-0089, Wuhan, China) and cultured in in DMEM low sodium bicarbonate basal medium (HAKATA, Shanghai, China). H9c2 cells were seeded into 96-well plates at a density of 6 × 10^3^ cells per well. Phenylephrine (PE) (Cat#2838, Tocris, Shanghai, China), celecoxib (S1261, Selleck, Shanghai, China) and S-Equol (100152, DAICEL, Shanghai, China) were dissolved in 0.01% DMSO, all of which were supplemented with 10 mM mother liquor. After 24 h of cell attachment, the cells were first divided into eight groups and treated for 48 h: the control group; 100 μM PE + 6 concentrations of S-Equol (0, 0.1, 0.5, 1, 5, and 10 μM); and the 100 μM PE + 50 μM celecoxib group. The eight groups of cells were then treated with a cell counting kit 8 (CCK-8) solution (MeilunBio, Liaoning, China) for 2 h. The absorbance of H9c2 cells at a wavelength of 450 nm was measured via a microplate reader (Bio-Rad, Hercules, CA, USA) to evaluate the effect of S-Equol on the PE-induced proliferative activity of H9c2 cells. For the subsequent WGA staining and western blot assays, H9c2 cells were divided into four groups for 48 h: control, 100 μM PE, 100 μM PE + 1 μM S-Equol, and 100 μM PE + 50 μM celecoxib.

### Wheat germ agglutinin (WGA) staining

H9c2 cells were seeded into four glass-bottom cell culture dishes at a density of 1 × 10^5^ cells/well, and the cells were dosed in groups after 24 h of attachment. After 48 h of intervention, the cells were washed once with PBS prewarmed at 37 °C, fixed with 4% PFA for 15 min at room temperature, washed once with PBS, incubated with Wheat Germ Agglutinin (W32464, Invitrogen™, USA) for 15 min at room temperature in the dark, washed with PBS three times, and then photographed with a laser-scanning confocal microscope at 555 nm.

### Western blot analysis

Protein extracts from cells were prepared via RIPA buffer containing anti-protease and anti-phosphatase reagents. The extracted protein concentration was determined via a BCA protein quantitation assay kit (P0012, Beyotime, Shanghai, China). A total of 20 μg of lysate was loaded onto SDS‒PAGE gels and transferred to PVDF membranes (Merck Millipore, GER). After being blocked with 2.5% bovine serum albumin (BSA) for 1 h, the membranes were incubated with primary antibodies (IL17A, 1:1000, A0688, ABclonal, Wuhan, China; PTGS2, 1:2000, CY8852, Abways, Shanghai, China) at 4 °C overnight. The membranes were subsequently rinsed 3 times with TBST and the secondary antibody goat anti-rabbit IgG-HRP (1:5000, SGRb1000H, NANOLAB, Shanghai, China) for 1 h. The bands were visualized via an electrochemiluminescence (ECL) kit (WBULS0500; EMD Millipore) and exposed with an ultra-high sensitivity chemiluminescence imaging machine (FluorChem®E, NatureGene Corp, USA). Finally, ImageJ software was used to analyze the gray values of the bands.

### Statistical analysis

The data are expressed as the means ± standard deviations (SDs). Statistical analyses were performed via GraphPad Prism 10.1.2. The differences between two groups or among many groups were analyzed with one-way analyses of variance. *P* < 0.05 was considered statistically significant.

## Results

### Acquisition of gut metabolites and potential targets

**Fig. 1:**
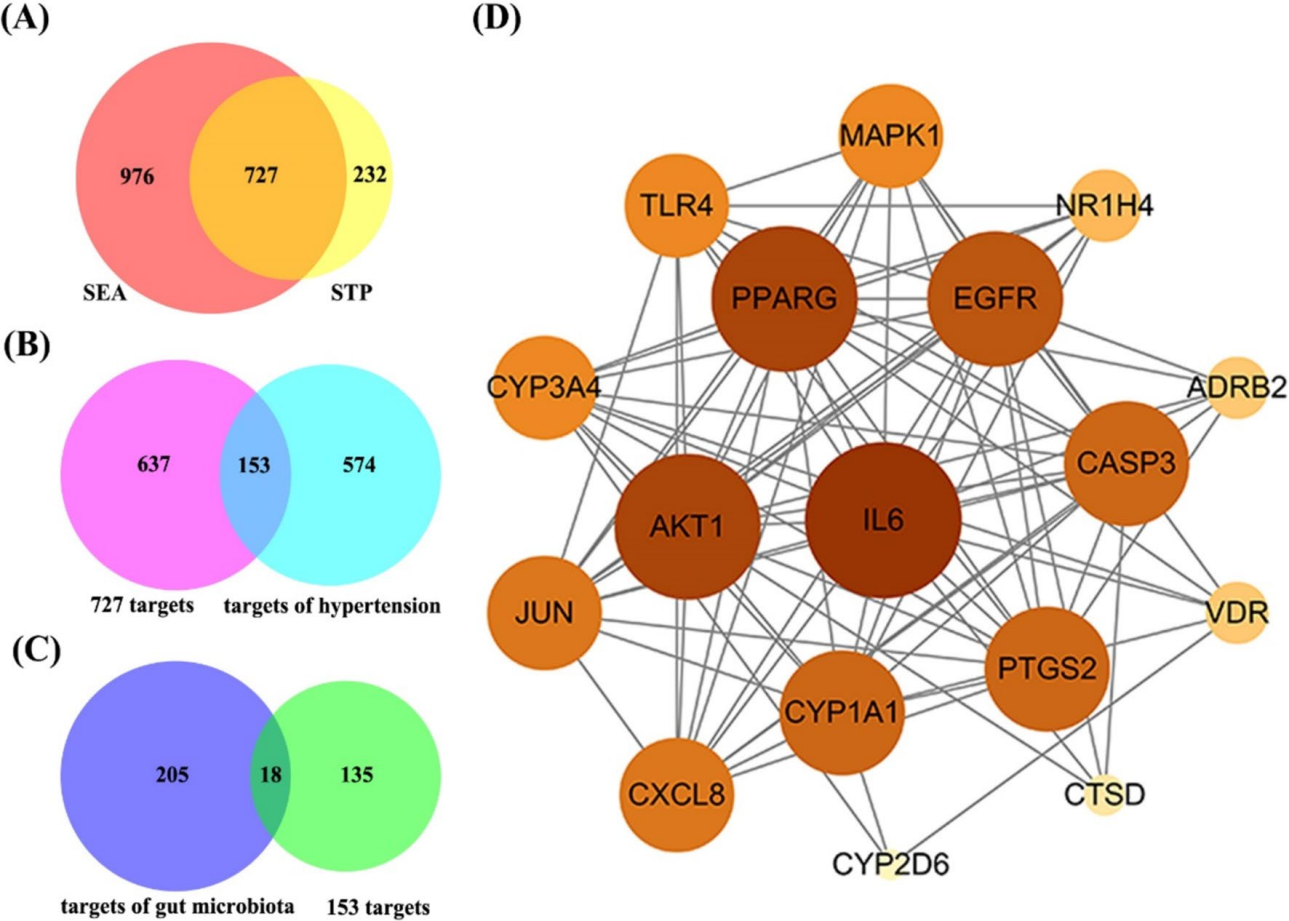
**A** The number of overlapping 727 targets between the SEA and STP database. **B** The number of overlapping 153 targets among the 727 targets and hypertension-related targets. **C** The 18 overlapping targets among the 153 targets and targets of the gut microbiota. **D** PPI network

We obtained 208 metabolites and 223 targets from the gutMGene v1.0 database. These metabolites and targets are currently considered to be therapeutically effective components of the gut microbiota. We imported the canonical SMILES of metabolites into the SEA and STP databases and obtained 1703 and 959 targets, respectively. Finally, 727 overlapping targets were identified (Fig. 1A).

### Identification of 18 core targets of gut metabolites involved in hypertension

After screening and deduplication, 789 hypertension-related targets were obtained from the GeneCards, OMIM, and TTD databases. There were 153 overlapping targets between the 727 and 789 hypertension-related targets (Fig. 1B). Eighteen overlapping targets were obtained between 153 and 223 gut microbiota targets as core targets for the gut metabolites that act on hypertension (Fig. 1C).

### PPI network and enrichment analysis

The PPI network consisted of 17 nodes and 85 edges out of 18 core targets (Fig. 1D). ADRAB2 has no connection with the other 17 nodes. The size of the nodes in the PPI network depends on the degree of the value. IL6, AKT1, and PPARG are important targets of the network.

**Fig. 2:**
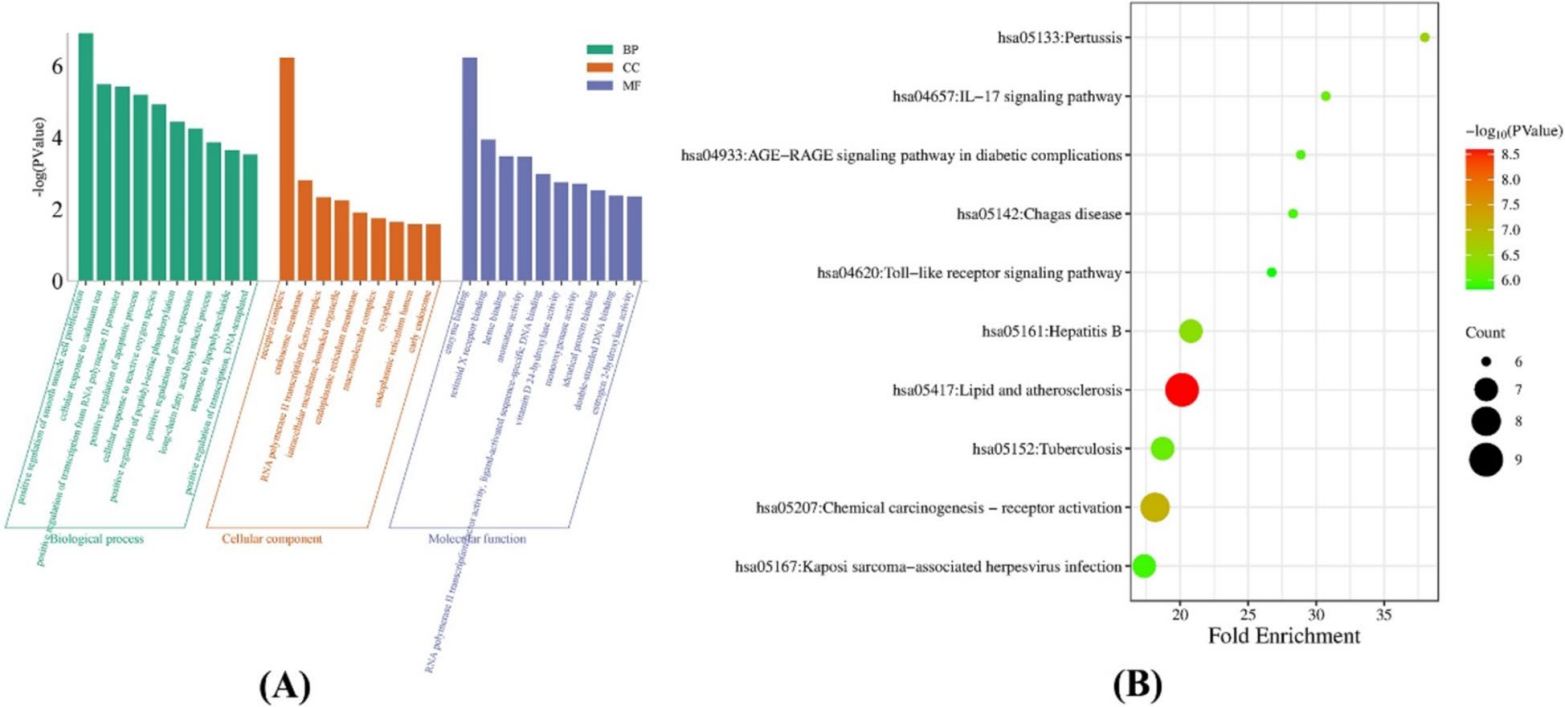
**A** GO analysis. **B** KEGG enrichment analysis

Eighteen core targets were entered into the DAVID database for GO and KEGG enrichment analysis. When *p* < 0.05, GO enrichment analysis revealed 91, 9, and 24 items for BP, CC, and MF, respectively (Fig. 2A). KEGG enrichment analysis revealed 93 signaling pathways. Among the top ten signaling pathways with -log10(p) values (Fig. 2B), lipid and atherosclerosis, the IL-17 signaling pathway, the AGE-RAGE signaling pathway in diabetes, and the Toll-like receptor signaling pathway may be important pathways affecting hypertension.

### Identification of key components in the MMTS network

**Fig. 3:**
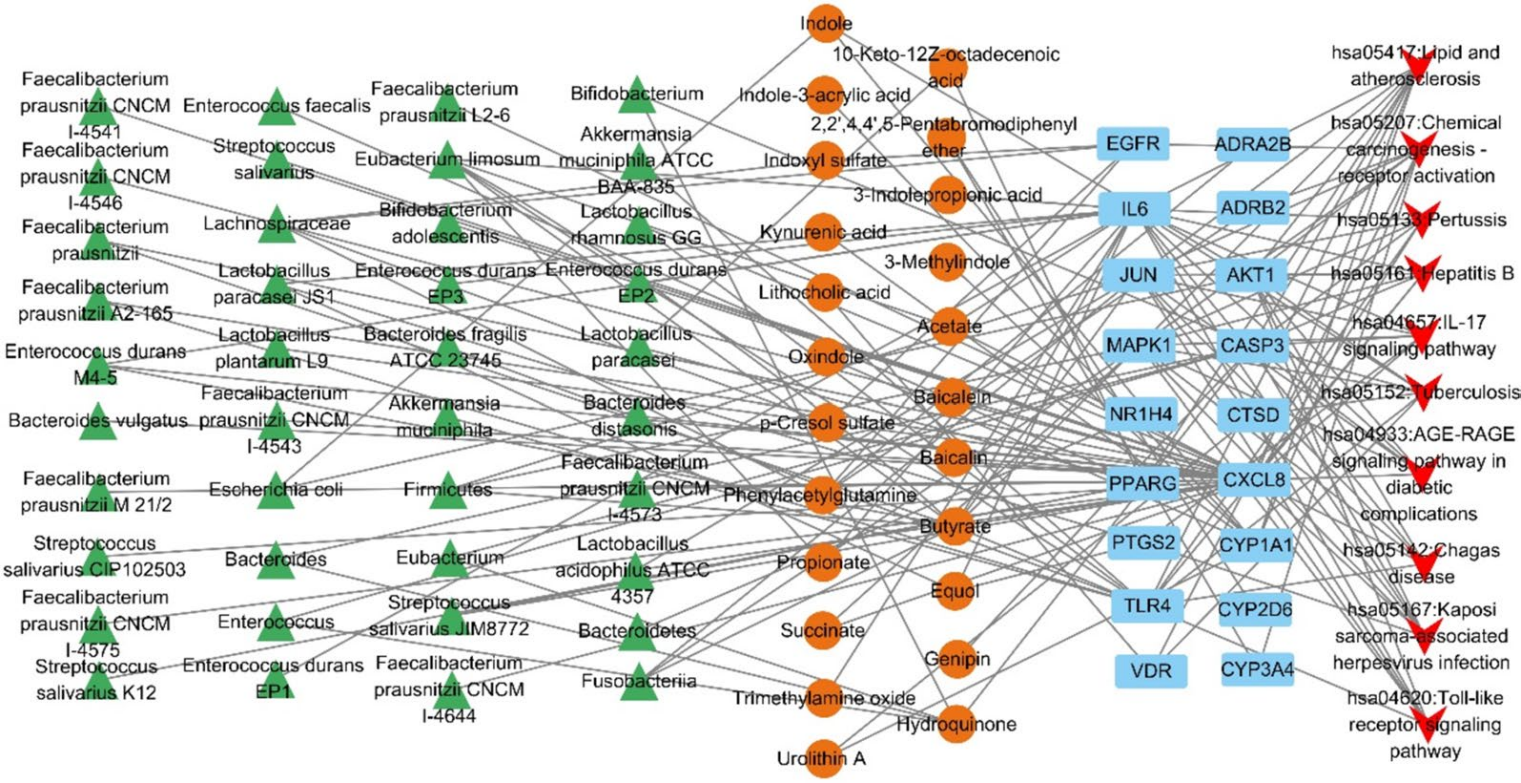
MMTS network. Green triangle: gut microbiota. Orange circle: gut metabolites. Blue rectangle: targets. Red quadrilateral: signaling pathways

The MMTS network was analyzed via Cytoscape 3.10.1, which consists of 91 nodes (40 microbiota, 23 metabolites, 18 targets, and 10 signaling pathways) and 170 edges (Fig. 3). Among them, lipids and atherosclerosis signaling pathway connects 18 metabolites and nine targets (IL6, JUN, CXCL8, CASP3, CYP1A1, MAPK1, AKT1, PPARG, and TLR4). The IL-17 signaling pathway connects 11 metabolites and six targets (IL6, JUN, CXCL8, CASP3, MAPK1, and PTGS2). The AGE-RAGE signaling pathway in diabetic complications is associated with 12 metabolites and six targets (IL6, JUN, CXCL8, CASP3, MAPK1, and AKT1). The TLR signaling pathway connects 10 metabolites and six targets (IL6, JUN, CXCL8, MAPK1, AKT1, and TLR4) (Table 1). We further analyzed 10 targets and 18 metabolites involved in these four pathways.

**Table 1:** Metabolites and core targets of important signaling pathways

Signaling pathways	Targets	Metabolites
hsa05417: Lipid and atherosclerosis	IL6, JUN, CXCL8, CASP3, CYP1A1, MAPK1, AKT1, PPARG, TLR4	10-keto-12Z-octadecenoic acid, 3-indolepropionic acid, 3-methylindole, acetate, baicalein, baicalin, butyrate, equol, genipin, hydroquinone, indole, indole-3-acrylic acid, kynurenic acid, oxindole, propionate, succinate, trimethylamine oxide, urolithin A
hsa04657: IL-17 signaling pathway	IL6, JUN, CXCL8, CASP3, MAPK1, PTGS2	3-indolepropionic acid, acetate, butyrate, equol, propionate, trimethylamine oxide, baicalein, baicalin, succinate, genipin, hydroquinone
hsa04933: AGE-RAGE signaling pathway in diabetic complications	IL6, JUN, CXCL8, CASP3, MAPK1, AKT1	3-indolepropionic acid, acetate, butyrate, equol, propionate, trimethylamine oxide, baicalein, baicalin, succinate, genipin, hydroquinone, indole
hsa04620: Toll-like receptor signaling pathway	IL6, JUN, CXCL8, MAPK1, AKT1, TLR4	3-indolepropionic acid, acetate, butyrate, equol, propionate, trimethylamine oxide, baicalein, baicalin, succinate, indole

### Results of the molecular docking test

Molecular docking involves the docking of a ligand and receptor in an active pocket, which is accompanied by a change in binding energy. A docking binding energy of less than 0 kcal·mol^−1^ indicates that the receptor and ligand can bind spontaneously. The lower the binding energy is, the greater the likelihood of binding, the more stable the binding conformation, and the greater the likelihood of a reaction. A docking binding energy of less than − 5 kcal·mol^−1^ indicates that there is good binding potential between the receptor and the ligand (Paquet and Viktor 2015).

**Fig. 4:**
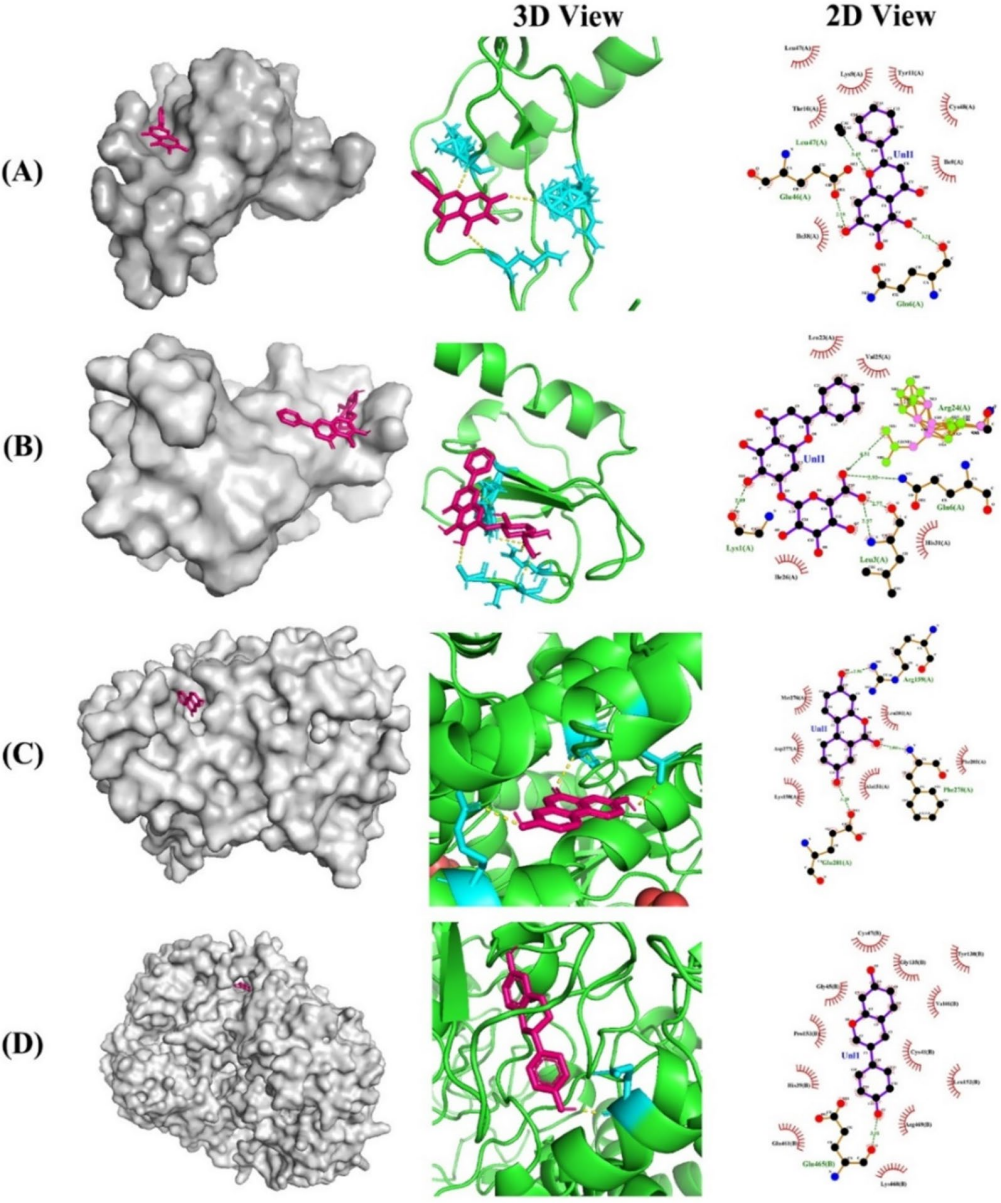
Molecular docking results of metabolites and core targets. **A** CXCL8-baicalein. **B** CXCL8-baicalin. **C** CYP1A1-urolithin A. **D** PTGS2-equol

We performed molecular docking analysis on 10 targets and 18 metabolites related to these four signaling pathways. The docking results revealed that 26 receptor‒ligand binding conformations have binding potential (Table 2), among which CXCL8-baicalein, CXCL8-baicalin, CYP1A1-urolithin A, and PTGS2-equol have strong binding potential. As shown in Fig. 4A, baicalein interacted with Gln6(A), Glu46(A), and Leu47(A) through hydrogen bonds in CXCL8, with a binding energy of − 7.7 kcal-mol^−1^. As shown in Fig. 4B, baicalin interacted with Lys1(A), Leu3(A), Gln6(A), and Arg24(A) through hydrogen bonds in CXCL8, with a binding energy of − 8.5 kcal-mol^−1^. As shown in Fig. 4C, urolithin A interacted with Phe278(A), Glu281(A), and Arg159(A) through hydrogen bonds in CYP1A1, with a binding energy of − 7.2 kcal-mol^−1^. As shown in Fig. 4D, equol interacted with Glu465(B) through hydrogen bonds in PTGS2, with a binding energy of − 8.8 kcal-mol^−1^.

**Table 2:** Molecular docking results of metabolites and core targets

Target	Target PDB ID	Target protein structure	Metabolites	PubChem ID	Metabolites 3D structure	Binding energy (kcal·mol^−1^)	Grid box center		
							X	Y	Z
IL6	1ALU		3-indolepropionic acid	3744		− 5.2	16	− 4.7	− 12.9
			Acetate	175		− 2.6	16	− 4.7	− 12.9
			Butyrate	104,775		− 3.2	16	− 4.7	− 12.9
			Equol	91,469		− 5.9	16	− 4.7	− 12.9
			Propionate	104,745		− 2.9	16	− 4.7	− 12.9
			Trimethylamine oxide	1145		− 2.4	16	− 4.7	− 12.9
JUN	5T01		Butyrate	104,775		− 3	16	− 4.7	− 12.9
CASP3	1NME		Genipin	442,424		− 5.1	30	104	8.5
			Hydroquinone	785		− 4.2	30	104	8.5
CXCL8	4XDX		Acetate	175		− 3	16	− 4.7	− 12.9
			Baicalein	5,281,605		− 7.7	16	− 4.7	− 12.9
			Baicalin	64,982		− 8.5	16	− 4.7	− 12.9
			Butyrate	104,775		− 4	16	− 4.7	− 12.9
			Succinate	160,419		− 4.8	16	− 4.7	− 12.9
CYP1A1	5JKV		3-methylindole	6736		− 5.8	83.5	55.5	48.5
			Indole	798		− 5.7	83.5	55.5	48.5
			Indole-3-acrylic acid	5,375,048		− 6.3	83.5	55.5	48.5
			Kynurenic acid	3845		− 6.6	83.5	55.5	48.5
			Oxindole	321,710		− 4.8	83.5	55.5	48.5
			Urolithin A	5,488,186		− 7.2	83.5	55.5	48.5
MAPK1	8AOJ		Butyrate	104,775		− 3	16	− 4.7	− 12.9
			Succinate	160,419		− 3.5	16	− 4.7	− 12.9
AKT1	1UNQ		Indole	798		− 3.5	22.6	41	40
PPARG	6T1S		10-keto-12Z-octadecenoic acid	24,970,825		− 5.7	3.5	− 9.5	27
TLR4	2Z62		Butyrate	104,775		− 3.7	16	− 4.7	− 12.9
PTGS2	5F19		Equol	91,469		− 8.8	14.4	46.9	13.1

### *Identification of drug-likeness properties *in silico

Four metabolites (baicalin, baicalein, urolithin A, and equol) were identified via ADME parameters in silico (Table 3). Baicalin violates Lipinski's rule, where the number of hydrogen bond acceptors (HBAs), number of hydrogen bond donors (HBDs), and topological polar surface area (TPSA) do not fulfill the drug-likeness requirement. The remaining three metabolites exhibit drug-like physicochemical properties. Therefore, we conclude that baicalein, urolithin A, and equol are likely to affect hypertension.

**Table 3:** Drug-likeness properties of the metabolites from the gut microbiota

Metabolites	Lipinski’s rule				Lipinski’s violations ≤ 1	Bioavailability score > 0.1	TPSA < 140 Å^2^
	MW ≤ 500 g/mol	HBA ≤ 10	HBD ≤ 5	MLOGP ≤ 5			
Baicalin	446.36	11	6	− 1.63	2	0.11	187.12
Baicalein	270.24	5	3	0.52	0	0.55	90.9
Urolithin A	228.2	4	2	1.68	0	0.55	70.67
Equol	242.27	3	2	2.2	0	0.55	49.69

MW: Molecular weight. MLOGP: Log *P*_o/w_

### Toxicological properties of the three metabolites

**Fig. 5:**
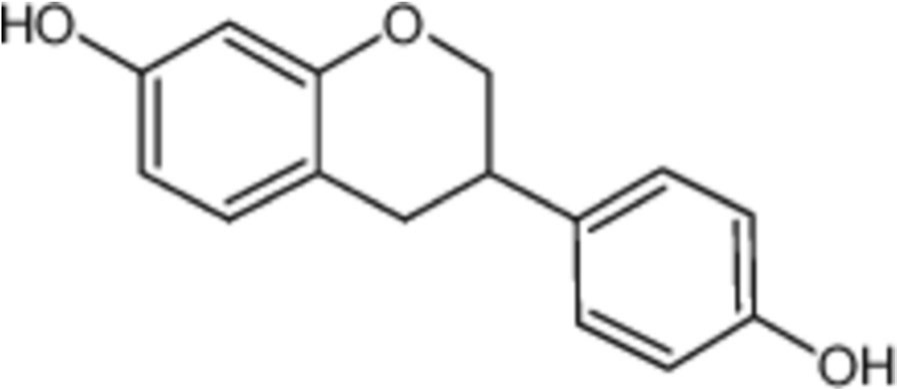
Chemical structure of equol

We evaluated the safety of these metabolites from a toxicological perspective. ADMElab was used to assess the toxicological profiles of the three metabolites (Table 4). On the basis of the toxicological predictions of the ADMElab platform, equol could be a promising metabolite for the treatment of hypertension (Fig. 5).

**Table 4:** Toxicological properties of the metabolites from the gut microbiota

Metabolites	hERG blockers	Human hepatotoxicity (H-HT)	AMES Toxicity	Respiratory toxicity	Rat oral acute toxicity	Drug induced liver injury (DILI)	Skin sensitization	LD50 of acute toxicity (mg/kg)
Baicalein	Non-blockers	Negative	Negative	Negative	Negative	Positive	Non-sensitizer	2.714
Urolithin A	Non-blockers	Negative	Negative	Negative	Negative	Positive	Non-sensitizer	2.349
Equol	Non-blockers	Negative	Negative	Negative	Negative	Negative	Non-sensitizer	2.204

### Effect of S-Equol on the proliferation of PE-induced H9c2 cells

**Fig. 6:**
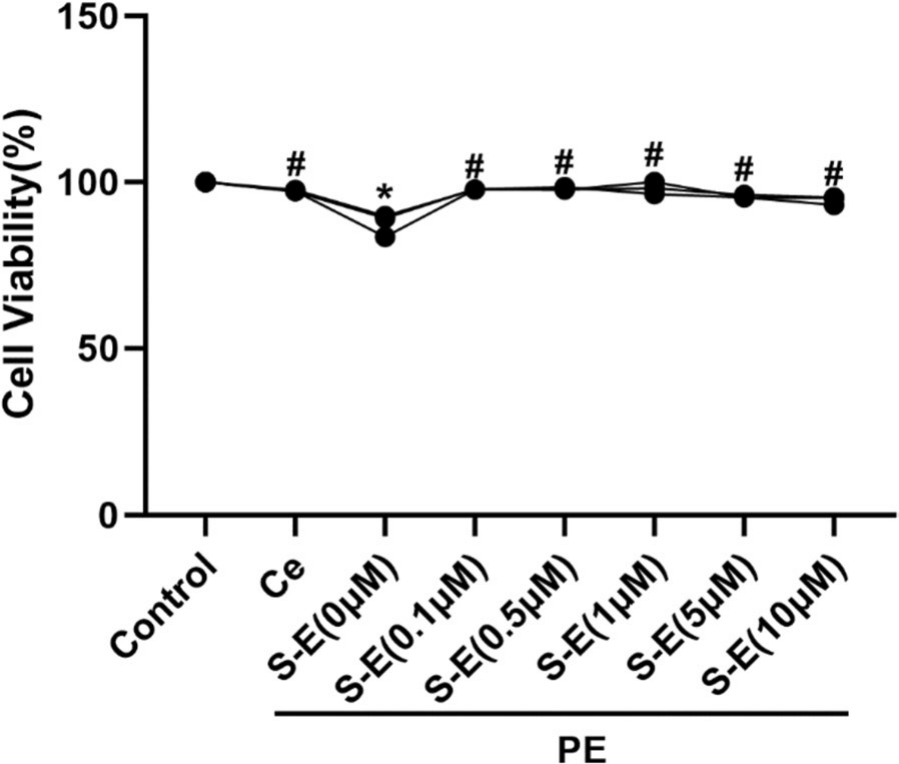
Cell viability assessment via the CCK8 assay. PE: Phenylephrine. Ce: Celecoxib. S‒E: S‒Equol

We explored the effects of different concentrations of S-equol on the PE-induced proliferation of H9c2 cells and compared them with those of the PTGS2-specific inhibitor celecoxib. Compared with the S-Equol 0 μM group, the treatment with S-Equol 0.1, 0.5, 1, 5 and 10 μM significantly increased cell viability (P < 0.05). The cell viability of the 0.1, 0.5, 1, 5, and 10 μM S-Equol groups did not differ from that of the control and celecoxib groups (P < 0.05). Among the groups, the S-Equol 1 μM group had the highest cell viability of 98.19%, which was greater than that of the celecoxib group (97.40%). Ultimately, 1 μM was determined to be the optimal concentration of S-equol (Fig. 6).

### S-Equol attenuated PE-induced hypertrophy in H9c2 cells

**Fig. 7:**
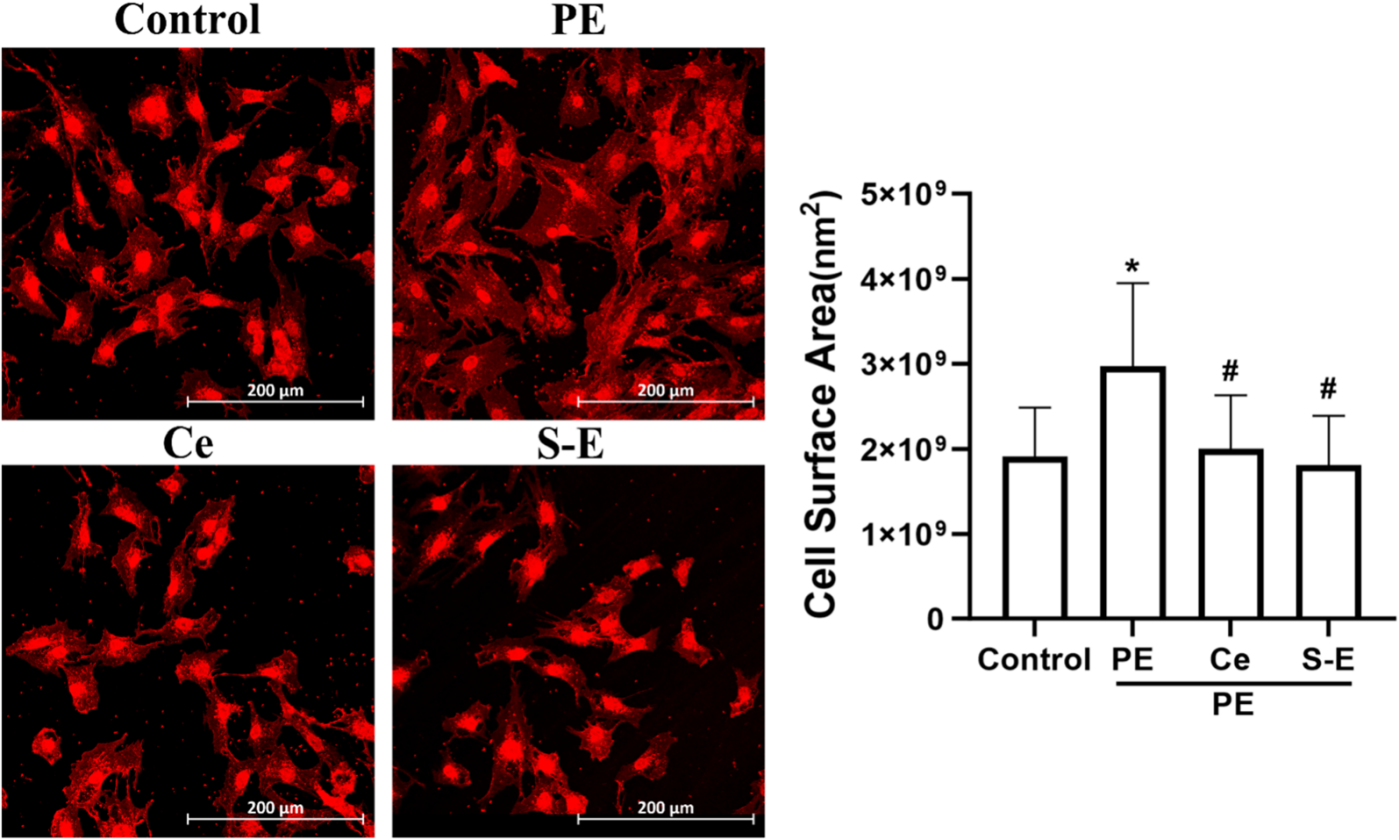
WGA staining (*n* = 3). PE: Phenylephrine. Ce: Celecoxib. S‒E: S‒Equol

We further examined the effects of S-equol on cell hypertrophy. Compared with the PE group, the S-Equol treatment significantly reduced the cell surface area (P < 0.05), and the cell surface area of the S-Equol group was smaller than that of the celecoxib group (P > 0.05) (Fig. 7). Therefore, S-equol inhibits cell hypertrophy.

### S-Equol reduced the protein levels of IL-17A and PTGS2 in PE-induced H9c2 cells

**Fig. 8:**
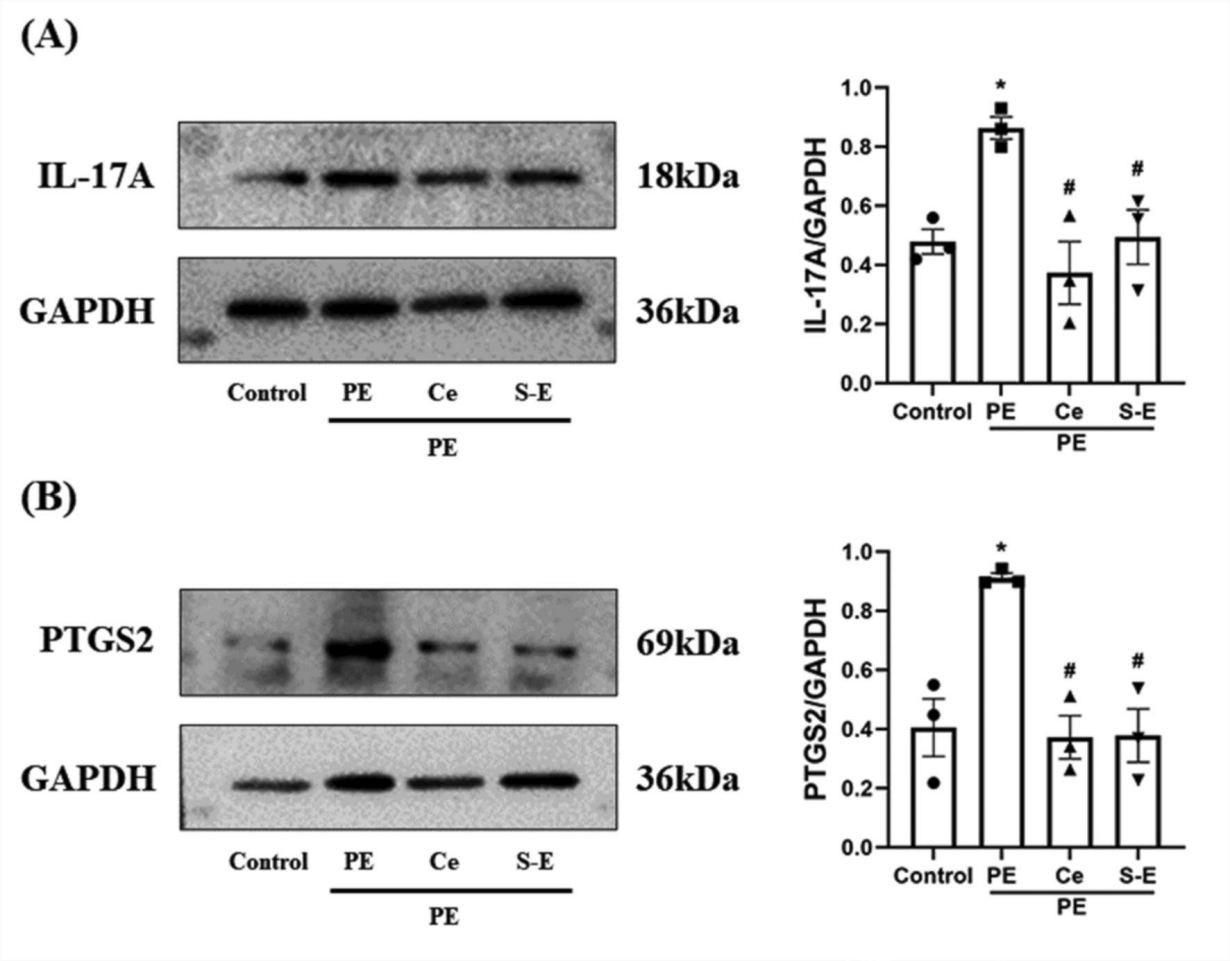
S-Equol reduced the IL-17A and PTGS2 protein levels in PE-induced H9c2 cells. **A** IL-17A protein content (*n* = 3). **B** PTGS2 protein content (*n* = 3). PE: Phenylephrine. Ce: Celecoxib. S‒E: S‒Equol

On the basis of the results of network pharmacology and molecular docking, it was necessary to verify the effects of S-equol on the IL-17 signaling pathway and PTGS2. IL-17A is a major inflammatory factor located upstream of the IL-17 signaling pathway. Therefore, we performed western blotting to detect the protein expression levels of IL-17A and PTGS2. Western blotting revealed that the relative protein expression levels of IL-17A and PTGS2 significantly increased under PE treatment, whereas S-Equol treatment reversed this change (P < 0.05) (Fig. 8).

## Discussion

Hypertension can cause damage to target organs, such as the heart, brain, kidneys, and blood vessels, and has a significant negative impact on people's lives (Kjeldsen. 2018). However, current antihypertensive drugs may have side effects. O'Donnell et al. reported a link between the gut metabolites and hypertension (O'Donnell et al. 2023). In this study, we applied network pharmacology and molecular docking techniques to explore the gut metabolites that may be associated with hypertension to discover new ways to treat hypertension.

We applied network pharmacology to suggest that equol may play an anti-hypertensive role by regulating the IL17 pathway. Interleukin-17A (IL-17A), the main inflammatory factor of the IL-17 signaling pathway, is produced by Th17 cells and is a key regulator of hypertension. It regulates blood pressure by decreasing endothelial nitric oxide (NO) production and increasing the production of reactive oxygen species (ROS) (Iwakura et al. 2008; Lee et al. 2019). By applying molecular docking techniques to simulate the possible binding sites of metabolites and related proteins, we concluded that the antihypertensive effect of equol was closely related to PTGS2. A meta-analysis of 7017 hypertensive patients revealed high PTGS2 expression in patients who did not receive antihypertensive therapy (Huan et al. 2015). PTGS2 inhibitors can lower blood pressure by inhibiting angiotensin-converting enzyme (ACE) activity (Mitchell et al., (2021). These findings validated the accuracy of the network pharmacology. Therefore, equol may act as a PTGS2 inhibitor for the treatment of hypertension.

Although molecular docking revealed that equol had good docking activity with PTGS2, the results were not experimentally verified and could not be applied. Therefore, we performed in vitro experiments to further elucidate the antihypertensive mechanism of equol. PTGS2 is located downstream of the IL-17 signaling pathway. The major upstream inflammatory factor, IL-17A, stimulates PTGS2 expression and increases NO production by regulating the activity of downstream inflammatory signaling pathways, such as the NF-κB and MAPK pathways, thus directly regulating blood pressure (Karbach et al. 2014; Bai et al. 2018). The PE-induced H9c2 cell model is considered a cell model that can be used to study stress overload-induced myocardial hypertrophy (Lu et al. 2023). Equol exists in the form of the diastereomers S-Equol and R-Equol, in which the intestinal bacteria produce only S-Equol (Setchell and Clerici 2010). Therefore, we used S-Equol as the research object and conducted in vitro experiments on a PE-induced H9c2 cell model to verify the effects of S-Equol on IL-17A and PTGS2 in the IL-17 signaling pathway. The experimental results showed that S-Equol significantly reduced the expression of IL-17A and PTGS2 in PE-induced H9c2 cells and reduced the H9c2 cell area. Thus, the antihypertensive effect of equol is related to the inhibition of the IL-17 signaling pathway and PTGS2.

**Fig. 9:**
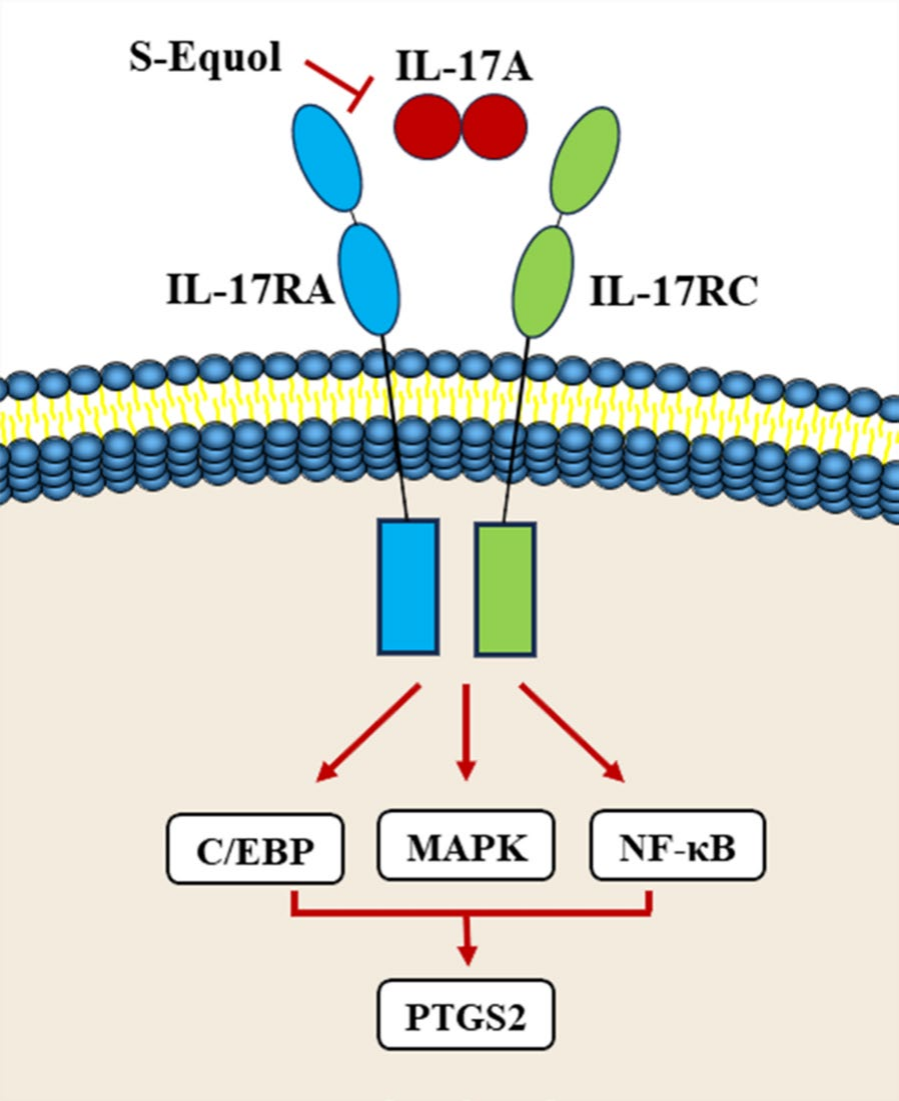
Mechanistic diagram

Previous studies have shown that the gut metabolite equol and its gut microbiota *Lactobacillus paracasei* JS1, which are closely associated with the MMTS network, have antihypertensive effects. A cohort study of 6,953 people revealed that the abundance of *Lactobacillus paracasei* was strongly negatively correlated with salt intake and blood pressure (Palmu et al. 2020). A 229-patient case‒control study revealed that high plasma equol concentrations help prevent prehypertension and hypertension (Lee et al. 2021). Liu TH reported that equol modulated blood pressure in DHRs by inhibiting angiotensin-converting enzyme (ACE) activity and increasing NO production (Liu and Tsai 2016). Our in vitro experiments complemented the antihypertensive mechanism of equol as a PTGS2 inhibitor to inhibit ACE activity and thus reduce blood pressure, as shown in previous studies (Fig. 9).

A systematic analysis revealed that equol has the potential to be developed into a new drug for the treatment of hypertension. Therefore, our results are valuable.

## Conclusion

In conclusion, using network pharmacology and molecular docking techniques, we investigated potential gut metabolites with antihypertensive properties. On the basis of computational drug similarity and toxicological analyses, we identified equol as a gut metabolite with promising antihypertensive potential. Through in vitro experiments, we subsequently demonstrated that equol exerts antihypertensive effects by inhibiting the IL-17 signaling pathway and its downstream target PTGS2. Future research should focus on validating the efficacy, safety, and mechanism of action of equol in hypertension via in vivo models, thereby providing a robust foundation for subsequent preclinical studies.

## Supplementary Information

Additional file 1.

## Data Availability

All data generated or analyzed during this study are included in this published article and its supplementary information files.

## Abbreviations

ACE: Angiotensin-converting enzyme
ACEI: Angiotensin-converting enzyme inhibitor
ADME: Absorption, distribution, metabolism, and excretion
ADRAB2: Alpha-2B adrenergic receptor
AKT1: RAC-alpha serine/threonine-protein kinase
Ang II: Angiotensin II
BF: Biological function
CARDIA: Coronary artery risk development in young adults
CASP3: Caspase-3
CC: Cellular component
Ce: Celecoxib
CVD: Cardiovascular disease
CXCL8: Interleukin-8
CYP1A1: Cytochrome P450 1A1
DAVID: The database for annotation, visualization, and integrated discovery
DHR: DOCA-salt-induced hypertensive rat
DILI: Drug-induced liver injury
DOCA: Deoxycorticosterone acetate
eNOS: Endothelial NO synthase
GABA: Gamma-aminobutyric acid
GO: Gene Ontology
HBA: Hydrogen-bond acceptor
HBD: Hydrogen-bond donor
H-HT: Human hepatotoxicity
IL-17: Interleukin-17
IL6: Interleukin-6
JUN: Transcription factor Jun
KEGG: Kyoto Encyclopedia of Genes and Genomes
LD50: Lethal dose 50
MAPK1: Mitogen-activated protein kinase 1
MF: Molecular function
MMTS: Microbiota–metabolites–targets–signaling pathways
MW: Molecular weight
NO: Nitric oxide
OMIM: Online Mendelian inheritance in man
PE: Phenylephrine
PPARG: Peroxisome proliferator-activated receptor gamma
PPI: Protein–protein interaction
PTGS2: Prostaglandin endoperoxide synthase 2
RCSB PDB: Research Collaboratory for Structural Bioinformatics Protein Data Bank
S-E: S-Equol
SCFA: Short-chain fatty acid
SEA: Similarity ensemble approach
SHR: Spontaneously hypertensive rat
SMILES: Simplified Molecular-Input Line-Entry System
STP: SwissTargetPrediction
TLR4: Toll-like receptor 4
TPSA: Topological polar surface area
TTD: Therapeutic target database
